# The Effect of Cerebellar Transcranial Direct Current Stimulation on Motor Learning: A Systematic Review of Randomized Controlled Trials

**DOI:** 10.3389/fnhum.2019.00328

**Published:** 2019-10-04

**Authors:** Nitika Kumari, Denise Taylor, Nada Signal

**Affiliations:** Health and Rehabilitation Research Institute, Auckland University of Technology, Auckland, New Zealand

**Keywords:** transcranial direct current stimulation, tDCS, cerebellum, motor learning, motor adaptation, skill learning

## Abstract

**Background:** Cerebellar transcranial direct current stimulation (ctDCS) appears to modulate motor performance in both adaptation and motor skill tasks; however, whether the gains are long-lasting is unclear.

**Objectives:** This systematic review aims to evaluate the effect of ctDCS with respect to different time scales of motor learning.

**Methods:** Ten electronic databases (CINAHL, MEDLINE, SPORT Discus, Scopus, Web of Science, Cochrane via OVID, Evidence-Based Reviews (EBM) via OVID, AMED: Allied and Complementary Medicine, PsycINFO, and PEDro) were systematically searched. Studies evaluating the effect of ctDCS compared to sham ctDCS on motor learning in healthy individuals were selected and reviewed. Two authors independently reviewed the quality of the included studies using the revised Cochrane's risk-of-bias tool. The results were extracted with respect to the time scale in which changes in motor performance were evaluated.

**Results:** Seventeen randomized controlled trials met the eligibility criteria of which 65% of the studies had a “high” risk-of-bias, and 35% had “some concerns.” These studies included data from 629 healthy participants. Of the studies that evaluated the effect of anodal ctDCS during and immediately after the stimulation, four found enhanced, three found impaired, and ten found no effect on gains in motor performance. Of the studies that evaluated the effect of anodal ctDCS after a break of 24 h or more, seven found enhanced, two found impaired, and one found no effect on gains in motor performance. Of the studies that evaluated the effect of cathodal ctDCS across a range of time scales, five found impaired, one found enhanced, and five found no effect on gains in motor performance.

**Conclusions:** In healthy individuals, anodal ctDCS appears to improve short to longer-term motor skill learning, whereas it appears to have no effect on gains in motor performance during and immediate after the stimulation. ctDCS may have potential to improve motor performance beyond the training period. The challenge of the motor task and its characteristics, and the stimulation parameters are likely to influence the effect of ctDCS on motor learning.

## Introduction

Motor learning is the set of processes associated with practice or experience, which lead to a relatively permanent change in skilled motor performance (Schmidt and Lee, 2011). This is fundamental for acquiring new motor skills, responding to dynamic environmental conditions and for re-learning lost motor skills after injury (Kitago and Krakauer, 2013). Repeated training or practice is required to acquire complex motor skills and achieve peak performance. Therefore, strategies which maximize performance and enhance the acquisition of motor skills have received considerable attention in motor learning and rehabilitation literature (Winstein et al., 2014).

Recently, the modulation of cortical and sub-cortical excitability through external means such as non-invasive brain stimulation has received increasing attention as a means to enhance performance during training (Banissy and Muggleton, 2013; Okano et al., 2015; Edwards et al., 2017). One such application is transcranial direct current stimulation (tDCS). tDCS involves the delivery of continuous, weak electric currents to the brain to alter the resting membrane potentials of neurons to influence excitability (Bolognini et al., 2009). There is growing consumer interest in the ability of tDCS to modulate brain activity. Halo Sport (2019) and Caputron (2019) are two examples of commercially available tDCS devices being marketed to sporting populations. The manufacturers make reference to research evidence which illustrates the efficacy of tDCS to enhance motor performance (Waters-Metenier et al., 2014; Ciechanski et al., 2017), including in sporting populations (Huang et al., 2019). Much of the tDCS research has focused on the primary motor cortex and pre-motor areas (Ammann et al., 2016); however, researchers are increasingly considering the cerebellum as a target (Block H. J. and Celnik, 2012; Ferrucci et al., 2016, 2019; Grimaldi et al., 2016). The cerebellum contributes to the control of both motor and non-motor behaviors, including learning, posture and balance, coordination, cognition, emotion, and language (Timmann and Daum, 2007; Manto et al., 2012; Perciavalle et al., 2013; Koziol et al., 2014; Mariën et al., 2014; Caligiore et al., 2017; Lang et al., 2017). The cerebellum has a particular role in error-based learning (Miall and Wolpert, 1996; Diedrichsen et al., 2005; Tseng et al., 2007). In error-based learning, sensory prediction errors; the difference between predicted sensory consequences of a movement command, and the resultant sensory feedback, are used to adjust the subsequent motor output (Miall and Wolpert, 1996; Wolpert and Flanagan, 2001; Izawa and Shadmehr, 2011). Furthermore, evidence from neurophysiological, neuroimaging and behavioral studies in animals and humans suggest that cerebellar activation varies with the type of motor task performed and the stage of motor learning (Doyon and Benali, 2005; Dayan and Cohen, 2011; Lohse et al., 2014). Given the importance of the cerebellum in error-based motor learning (Ito, 2000; De Zeeuw and Ten Brinke, 2015) and re-learning of motor skills after central nervous system injury (Small et al., 2002; Ward et al., 2003; Sokolov et al., 2017), transcranial direct current stimulation over the cerebellum (ctDCS) has been advocated as an alternative tDCS stimulation site to promote motor learning (Grimaldi et al., 2014; Celnik, 2015; Oldrati and Schutter, 2018).

In a laboratory setting, motor learning is often evaluated using two paradigms: motor adaptation or skill learning. Motor adaptation consists of a perturbation applied during the performance of a well-learnt motor skill, for example, perturbing limb trajectories during reaching. The learner adapts to the error induced by the perturbation rapidly over minutes to hours (adaptation). When the perturbation is removed, the adaptation is retained for a period of time (after-effects) and gradually wanes over time (de-adaptation) (Martin et al., 1996). However, with repeated exposure to the perturbation, learning is observed through rapid reductions in errors (Martin et al., 1996) and faster rates of adaptation on subsequent exposures (Kojima et al., 2004). In motor skill learning paradigms, learning is evaluated through exposure to a novel motor task. Motor learning is observed through the reduction of errors and performance improvement beyond baseline levels (Reis et al., 2009).

Motor learning occurs over distinct phases. There is the early (fast) learning in which improvements in performance are seen rapidly within a single training session (Doyon and Benali, 2005). In the later slow stage, further performance gains are seen across several sessions of practice (Dayan and Cohen, 2011). Progression from fast to slow learning depends on appropriate rest periods and subsequent sleep (Diekelmann et al., 2009), where gains in performance can be observed without the additional practice of the task (Dayan and Cohen, 2011). Changes in performance are initially transient in nature, but with extended practice, the performance of skilled behavior becomes less attention-demanding and skilled performance is possible even after long breaks (Doyon and Benali, 2005). For the purposes of this paper, the time scales of learning are represented as (1) long-term changes in performance measured after a break of 24 h or more; (2) short-term change in performance after a break of <24 h; (3) change in performance measured immediately after training; and (4) change in performance during training.

There is ample evidence indicating that ctDCS can modulate cerebellar activity at a neurophysiological level (Galea et al., 2009), less is known about its effect on behavioral outcomes (Block H. and Celnik, 2012). To date, the evidence for the efficacy of ctDCS has been limited to its ability to modulate motor performance (Oldrati and Schutter, 2018). A recent meta-analysis reported the effectiveness of anodal and cathodal ctDCS in modulating motor performance in healthy individuals in both motor adaptation and motor skills tasks (Oldrati and Schutter, 2018), however, a systematic understanding of how ctDCS contributes to different timescales of motor learning is still lacking (Grimaldi et al., 2014; van Dun et al., 2016). Therefore, the present systematic review aims to elucidate the effects of ctDCS on motor learning across different time scales in healthy individuals to determine if the documented gains in performance persist for a substantial period after training. This understanding will be useful in ascertaining the prospects of using ctDCS as a neuro-modulatory tool to augment motor learning in both elite performance in healthy individuals and following brain lesions in clinical populations.

## Methods

### Study Design

A systematic search and review of the literature were undertaken based on an *a priori* plan.

### Inclusion and Exclusion Criteria

Studies were included if they met all the following criteria: involved healthy individuals above the age of 18 years, delivered real or sham tDCS over the cerebellum, random assignment to groups, measured behavioral outcomes of change in motor performance, and appeared in peer-reviewed English-language journals. Studies that compared different stimulation areas in the brain were included if data from cerebellar stimulation could be extracted and viewed separately.

Studies were excluded if they were reviews, books, theses, conference papers, commentaries, letters; if the sample consisted of animals; if the motor skill learning task did not involve the use of upper and lower limb; or if ctDCS was applied in combination with another intervention.

### Information Sources

A search (July 2019) of the following databases was undertaken: CINAHL, MEDLINE, SPORT Discus, Scopus, Web of Science, Cochrane via OVID, Evidence-Based Reviews (EBM) via OVID, AMED: Allied and Complementary Medicine, PsycINFO, and PEDro. No limit was placed on the publication date. The search strategy (Supplementary File 1) included following key search terms: acquisition, motor performance, motor control, learning, adapt^*^, ctDCS, cerebellar stimulation, tDCS, transcranial direct current stimulation, non-invasive brain stimulation, noninvasive brain stimulation, direct current stimulation, cerebell^*^. The reference list of included studies, recent systematic reviews, and meta-analyses were also searched.

### Study Selection

Following duplicate removal, the first author (N.K.) reviewed the titles and abstracts of all remaining studies. If a decision to include an article could not be made based on the title and abstract review, the full text was reviewed. A second reviewer (N.S.) was consulted if eligibility was unclear and a consensus reached.

### Data Extraction

Data was extracted using a form developed from the Cochrane data extraction and assessment template (Higgins and Green, 2011). Extracted information included the study characteristics, ctDCS stimulation parameters, motor learning task description, outcome measures, and key findings.

### Assessment of Study Quality

The quality of the included studies was critically appraised using the revised Cochrane's risk-of-bias tool for randomized trials (RoB 2) (Sterne et al., 2019). Two reviewers (N.K. and N.S.) independently rated the studies with any disagreements being discussed until consensus was reached. The revised Cochrane's risk-of-bias tool evaluates the methodological quality of the studies in relation to trial design, conduct, and reporting. Based on the answers to a series of signaling questions within five domains (randomization process, deviations from the intended interventions, missing outcome data, measurement of the outcome, and selection of the reported results), the studies were considered to have “low” or “high” risk-of-bias or “some concerns.” For randomized crossover trials signaling questions on carryover effect were additionally assessed. The overall risk-of-bias judgment for each study was categorized according to the revised Cochrane's risk-of-bias guidelines (Sterne et al., 2019).

## Results

### Search Results

**Figure 1:**
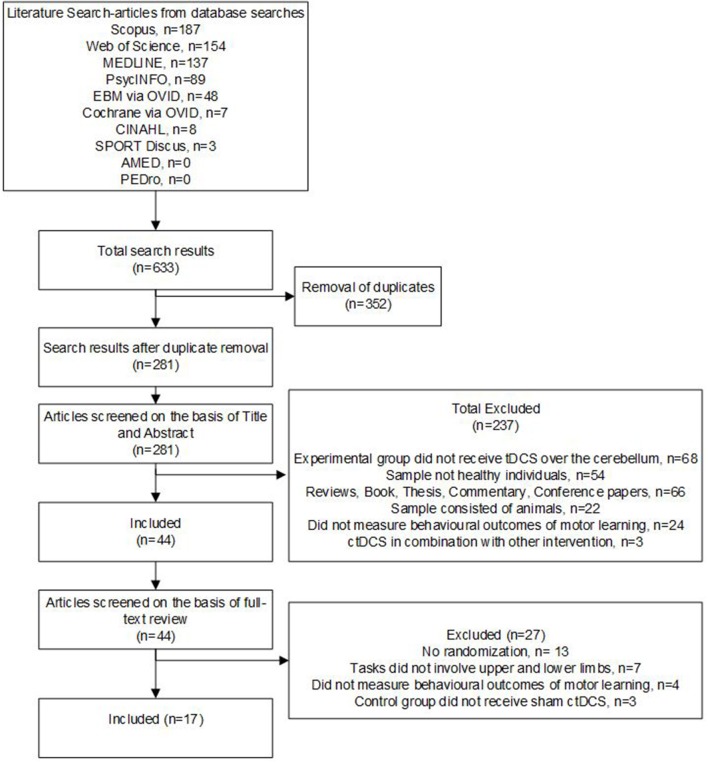
Flow chart showing the study selection process and results.

The electronic search retrieved 633 studies, which was reduced to 281 following duplicate removal. Title and abstract review excluded 237 studies which did not meet the eligibility criteria. On full-text review, a further 31 studies were excluded for reasons outlined in Figure 1.

Seventeen RCTs met the criteria for inclusion in this systematic review. No additional studies met the inclusion criteria upon searching the reference list of the included studies. The included studies constituted a total of 629 participants with a mean age between 18 and 69 years. Only two studies had participants above the age of 40 years (Panouillères et al., 2015; Samaei et al., 2017). Random allocation of participants was in either a parallel (*n* = 14) (Jayaram et al., 2012; Dutta et al., 2014; Panouillères et al., 2015; Ehsani et al., 2016; Panico et al., 2016; Taubert et al., 2016; Yavari et al., 2016; Samaei et al., 2017; Liew et al., 2018; Poortvliet et al., 2018; Summers et al., 2018; Jackson et al., 2019; Jongkees et al., 2019; Mamlins et al., 2019) or crossover design (*n* = 3) (Shah et al., 2013; Fernandez et al., 2017; Foerster et al., 2017), with 349 participants receiving real ctDCS. Refer to Table 1 (study characteristics).

**Table 1:** Characteristics of included studies.

**References**		**Sample size;** **mean age (years) ± SD**	**ctDCS stimulation type**	**Task**	**Training sessions**	**Outcome measure**	**Results**			
							**≥24 h**	**<24 h**	**IA**	**D**
Jayaram et al., 2012		40 (A = 8, C = 8, A = 8, C = 8, S = 8); 27, 20–33	A, C, and S	Adaptation: split-belt treadmill walking task	Single	Step length symmetry: rate, amount	NT	NT	A: X C: X	A: + C: –
Shah et al., 2013		8 (A = 8, C = 8, S = 8); 18–26	A, C, and S	Skill: ankle tracking task	Single for each condition	Normalized accuracy index	NT	A: + C: +	NT	NT
Dutta et al., 2014		8 (A = 4, S = 4); 24–36	A and S	Skill: myoelectric visual pursuit task	Single	Normalized response latency; tracking accuracy: mean absolute error	NT	NT	NT	–
Panouillères et al., 2015		53 (A = 26, S = 27); Old: 63.2 ± 7.5 Young: 22.5 ± 3.1	A and S	Adaptation: visuomotor rotation task	Single	Angular error	NT	X	NT	X
Yavari et al., 2016		29 (A = 10, C = 10, S = 9); 24 ± 5	A, C, and S	Adaptation: visuomotor adaptation task	Single	Reach angles; perception of hand position; mean reach direction	NT	NT	NT	A: + C: –
Ehsani et al., 2016		39 (A = 20, S = 19); 22.77 ± 1.32	A and S	Skill: serial response time task	Single	Response time (RT); number of errors (ER)	RT: + ER: +	RT: X ER: +	NT	RT: X ER: +
Taubert et al., 2016		41 (A = 14, C = 12, S = 15); 27 ± 3	A, C, and S	Adaptation: force field adaptation task	Single	Reaching error; set-break forgetting	A: – C: X	NT	NT	A: – C: X
Panico et al., 2016		26 (C = 13, S = 13); 21.57 ± 2.33	C and S	Adaptation: visuomotor rotation task	Single	Error; Error rate; Time course of stimulation effect on error	NT	NT	NT	–
Fernandez et al., 2017		14 (C = 14, S = 14); 28.93 ± 4.59	C and S	Adaptation: spatio-temporal gait task	Single for each condition	SD of stride length and step time	NT	NT	–	NT
Samaei et al., 2017		30 (A = 15, S = 15); 68.70 ± 5.28	A and S	Skill: serial reaction time task	Single	Response time (RT); number of errors (ER)	RT: + ER: X	RT: + ER: X	NT	RT: X ER: X
Foerster et al., 2017		15 (A = 15, C = 15, S = 15); 21–24	A, C, and S	Adaptation: balance Control	Single for each condition	Overall stability index (OSI)	NT	NT	A: X C: –	NT
Poortvliet et al., 2018		28 (A = 14, S = 14); 25.64 ± 3.82	A and S	Adaptation: postural adaptation	Single	Postural steadiness: center of pressure displacement; SD; total path length	NT	NT	+	NT
Summers et al., 2018		14 (A = 7, S = 7); 28.8 ± 10.5	A and S	Skill: finger tracking task	Single	Tracking accuracy index	NT	NT	X	X
Liew et al., 2018		31 (A:16, S: 15), NG	A and S	Adaptation: visuomotor adaptation task	Single	Hand endpoint angle: target error (E); reaction time (RcT)	NT	NT	E: X RcT: X	E: X, RT:X
		19 (A:10, S:9), NG	A and S	Adaptation: visuomotor adaptation task	Single	Hand endpoint angle: target error	NT	NT	X	X
Jongkees et al., 2019		72 (A = 24, C = 24, S = 24); A: 19.8 ± 1.6, C: 19.5 ± 1.5, S: 19.3 ± 1.8	A, C, and S	Skill: serial reaction time task	Single	Percentage accuracy (ACC); reaction time (RcT)	A: ACC-X, RT- –; C: ACC-X, RT: X	NT	NT	A: ACC-X, RT- –; C: ACC-X, RT: X
Jackson et al., 2019		42 (A = 21, S = 21); 25 ± 3.9	A and S	Skill: overhand throwing task	Single	Endpoint error: total (T); online (On) and offline (Of) learning	T: +, Of: X	On: +	NT	NT
Mamlins et al., 2019	I	30 (A = 10, C = 10, S = 10); 24.1 ± 2.3	A, C, and S	Adaptation: force field adaptation task	Single	Maximum error (extent and rate of learning);	NT	NT	A:X, C:X	A:X, C:X
		30 (A = 10, C = 10, S = 10); 24.1 ± 2.3				Perpendicular velocity			A:X, C:X	A:X, C:X
	II	30 (A = 10, C = 10, S = 10); 22.3 ± 3.1	A, C and S	Adaptation: visuomotor adaptation task	Single	Angular end point error (extent and rate of learning)	NT	NT	A:X, C:X	A:X, C:X
		30 (A = 10, C = 10, S = 10); 22.3 ± 3.1							A:X, C:X	A:X, C:X
Summary total		*n* = 629	A = 15 C = 9	Adaptation = 10 Skill = 7			A = 5 C = 2	A = 5 C = 1	A = 6 C = 3	A = 11 C = 5

*I, experiment 1; II, experiment 2; IA, immediately after; D, during the intervention; A, anodal ctDCS; C, cathodal ctDCS; S, sham ctDCS; NT, not tested; +, enhanced; –, impaired; X, no effect; SD, standard deviation; NG, not given*.

**Figure 2:**
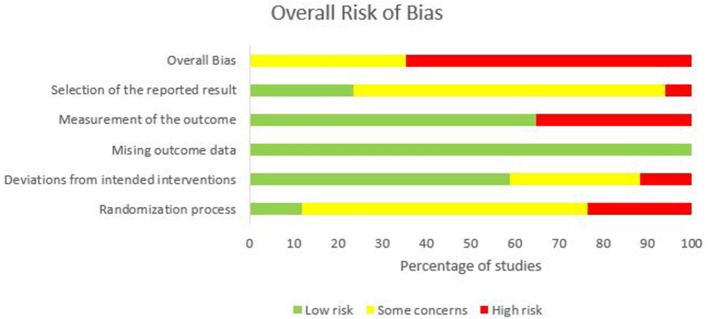
Overall risk-of-bias judgments for each domain.

One of the seventeen studies, six had “some concerns” (Shah et al., 2013; Ehsani et al., 2016; Fernandez et al., 2017; Samaei et al., 2017; Poortvliet et al., 2018; Jackson et al., 2019), and eleven had “high” risk-of-bias (Jayaram et al., 2012; Dutta et al., 2014; Panouillères et al., 2015; Panico et al., 2016; Taubert et al., 2016; Yavari et al., 2016; Foerster et al., 2017; Liew et al., 2018; Jongkees et al., 2019; Mamlins et al., 2019). Studies having “some concerns” were due to failure to explicitly report on the randomization process and trial registration or pre-specified statistical analysis plan. Studies having a “high” risk-of-bias was due to differences in baseline characteristics between the intervention groups suggesting issues with the randomization process, lack of information on blinding of the outcome assessor, the bias in the selection of reported results, and insufficient time for washout of carry-over effects. Refer to Figure 2, Supplementary File 2.

### ctDCS Intervention

The type of ctDCS stimulation varied across the studies. Eight studies applied anodal ctDCS (Dutta et al., 2014; Panouillères et al., 2015; Ehsani et al., 2016; Samaei et al., 2017; Liew et al., 2018; Poortvliet et al., 2018; Summers et al., 2018; Jackson et al., 2019), two cathodal ctDCS (Panico et al., 2016; Fernandez et al., 2017), and the remaining seven applied both anodal and cathodal stimulation (Jayaram et al., 2012; Shah et al., 2013; Taubert et al., 2016; Yavari et al., 2016; Foerster et al., 2017; Jongkees et al., 2019; Mamlins et al., 2019).

All studies investigated the effects of a single session of ctDCS. In the majority of studies (*n* = 9) stimulation was delivered *during* the training of a motor task (Jayaram et al., 2012; Shah et al., 2013; Dutta et al., 2014; Ehsani et al., 2016; Taubert et al., 2016; Yavari et al., 2016; Samaei et al., 2017; Summers et al., 2018; Jongkees et al., 2019). In three studies stimulation was delivered *prior* to the training of the task (Fernandez et al., 2017; Foerster et al., 2017; Poortvliet et al., 2018) and in the remaining five studies ctDCS was delivered just *prior to in conjunction with task training* (Panouillères et al., 2015; Panico et al., 2016; Liew et al., 2018; Jackson et al., 2019; Mamlins et al., 2019). The stimulation duration ranged between 8 and 30 min.

In tasks involving the upper limb, the stimulation was predominantly applied to the lateral cerebellum (*n* = 11) with respect to the training limb, ipsilaterally (*n* = 10) (Shah et al., 2013; Panouillères et al., 2015; Ehsani et al., 2016; Panico et al., 2016; Taubert et al., 2016; Yavari et al., 2016; Samaei et al., 2017; Liew et al., 2018; Jackson et al., 2019; Mamlins et al., 2019), or contralaterally (*n* = 1) (Dutta et al., 2014). Two studies applied the stimulation to the bilateral cerebellar hemispheres (Summers et al., 2018; Jongkees et al., 2019). Four studies investigated the effect of ctDCS on a bilateral task by placing the target electrode centrally (Poortvliet et al., 2018) or with respect to the dominant limb (Jayaram et al., 2012; Fernandez et al., 2017; Foerster et al., 2017). The return electrode was placed on the forehead (Dutta et al., 2014; Poortvliet et al., 2018), buccinator muscle (Jayaram et al., 2012; Shah et al., 2013; Taubert et al., 2016; Yavari et al., 2016; Fernandez et al., 2017; Summers et al., 2018), or upper limb (Panouillères et al., 2015; Ehsani et al., 2016; Panico et al., 2016; Foerster et al., 2017; Samaei et al., 2017).

ctDCS was delivered at a current density of 0.13 mA/cm^2^ (*n* = 1) (Shah et al., 2013), 0.08 mA/cm^2^ (*n* = 10) (Jayaram et al., 2012; Ehsani et al., 2016; Panico et al., 2016; Taubert et al., 2016; Yavari et al., 2016; Foerster et al., 2017; Samaei et al., 2017; Liew et al., 2018; Jackson et al., 2019; Mamlins et al., 2019), 0.06 mA/cm^2^ (*n* = 2) (Panouillères et al., 2015; Fernandez et al., 2017), or 0.03 mA/cm^2^ (*n* = 4) (Dutta et al., 2014; Poortvliet et al., 2018; Summers et al., 2018; Jongkees et al., 2019). Full details of the stimulation parameters are shown in Table 2.

**Table 2:** Stimulation parameters.

**References**	**ctDCS delivery**	**Electrode location**		**Electrode size (cm**^****2****^**)**		**Intensity (mA)**	**Density (mA/cm^**2**^)**	**ctDCS duration**	
		**Target**	**Return**	**Target**	**Return**			**Real (min.)**	**Sham (min.)**
Jayaram et al., 2012	During the task	Lateral cerebellar hemisphere, I/L and C/L to DL	Buccinator, I/L and C/L to DL	25	25	2	0.08	15	0.5
Shah et al., 2013	During the task	Left cerebellar hemisphere, I/L to TL	Left buccinator, I/L to TL	8	35	1	0.13	15	0
Dutta et al., 2014	During the task	Left cerebellar hemisphere, C/L to TL	Forehead above the right supraorbital ridge, I/L to TL	35	35	1	0.03	15	0.17
Panouillères et al., 2015	Prior + during the task	Right cerebellar hemisphere, I/L to TL	Left trapezius, C/L to TL	35	35	2	0.06	17	0.5
Yavari et al., 2016	During the task	Right cerebellar hemisphere, I/L to TL	Right buccinator I/L, to TL	25	25	2	0.08	15	0.5
Ehsani et al., 2016	During the task	Right cerebellar hemisphere, I/L to TL	Right deltoid, I/L to TL	25	25	2	0.08	20	1
Taubert et al., 2016	During the task	Right cerebellar hemisphere, I/L to TL	Right buccinator, I/L to TL	25	25	2	0.08	20	0.5
Panico et al., 2016	Prior + during the task	Right cerebellar hemisphere, I/L to TL	Right deltoid, I/L to TL	25	25	2	0.08	21	0.5
Fernandez et al., 2017	Prior to the task	Right cerebellar hemisphere, I/L to DL	Right buccinator, I/L to DL	35	35	2	0.06	20	0
Samaei et al., 2017	During the task	Right cerebellar hemisphere, I/L to TL	Right deltoid, I/L to TL	25	25	2	0.08	20	0.5
Foerster et al., 2017	Prior to the task	Right cerebellar hemisphere, I/L to TL	Right deltoid, I/L to TL	25	25	2	0.08	A:13C: 9	0.5
Poortvliet et al., 2018	Prior to the task	Ventral, dorsolateral aspects of the cerebellum and the cerebellar vermis	Centrally on the forehead	35	100	1	0.03	20	0.67
Summers et al., 2018	During the task	BL cerebellar hemisphere	Buccinator IL to TL	70	35	2	0.03	30	0.5
Liew et al., 2018	Prior + during the task	Right cerebellar hemisphere, I/L to TL	Buccinator IL to TL	25	25	2	0.08	>25	0.5
Jongkees et al., 2019	During the task	BL cerebellar hemisphere	BL mastoid	35	35	1	0.03	20	0.25
Jackson et al., 2019	Prior + during the task	Right cerebellar hemisphere, I/L to TL	Buccinator IL to TL	25	25	2	0.08	25	0.5
Mamlins et al., 2019	I: During, Prior + during	I: Right cerebellar hemisphere, I/L to TL	I: Buccinator IL to TL	I: 25	I: 25	I: 2	I: 0.08	I: 10.36 (0.12), 13.81 (0.19)	I: 1
	II: During, Prior + during	II: Right cerebellar hemisphere, I/L to TL	II: Buccinator IL to TL	II: 25	II: 25	II: 2	II: 0.08	II: 7.61 [0.17], 10.20 [0.16]	II: 1

*A, anodal ctDCS; C, cathodal ctDCS; I/L, ipsilateral; C/L, contralateral; TL, training limb; DL, dominant limb; BL, bilateral*.

### Motor Learning Tasks

Ten studies evaluated a motor adaptation task, and seven studies evaluated a motor skill task. The motor adaptation tasks included perturbation during visuomotor (Panouillères et al., 2015; Panico et al., 2016; Yavari et al., 2016; Liew et al., 2018; Mamlins et al., 2019), locomotor (Jayaram et al., 2012; Fernandez et al., 2017), reaching (Taubert et al., 2016), or postural control (Foerster et al., 2017; Poortvliet et al., 2018) tasks. Skill learning paradigms used serial reaction time task (Ehsani et al., 2016; Samaei et al., 2017; Jongkees et al., 2019), tracking (Shah et al., 2013; Dutta et al., 2014; Summers et al., 2018), or a throwing task (Jackson et al., 2019).

### Outcomes

Motor performance outcomes were measured based on error (*n* = 16) (Jayaram et al., 2012; Shah et al., 2013; Dutta et al., 2014; Panouillères et al., 2015; Ehsani et al., 2016; Panico et al., 2016; Taubert et al., 2016; Yavari et al., 2016; Foerster et al., 2017; Samaei et al., 2017; Liew et al., 2018; Poortvliet et al., 2018; Summers et al., 2018; Jackson et al., 2019; Jongkees et al., 2019; Mamlins et al., 2019), response latency (*n* = 1) (Dutta et al., 2014), response time (*n* = 2) (Ehsani et al., 2016; Samaei et al., 2017), reaction time (*n* = 2) (Liew et al., 2018; Jongkees et al., 2019), or movement variability (*n* = 2) (Fernandez et al., 2017; Poortvliet et al., 2018). Studies measured outcomes over a range of time scales including; after a break of 24 h or more post intervention (*n* = 5) (Ehsani et al., 2016; Taubert et al., 2016; Samaei et al., 2017; Jackson et al., 2019; Jongkees et al., 2019), after a break of <24 h post intervention (*n* = 5) (Shah et al., 2013; Panouillères et al., 2015; Ehsani et al., 2016; Samaei et al., 2017; Jackson et al., 2019), immediately after the intervention (*n* = 7) (Jayaram et al., 2012; Fernandez et al., 2017; Foerster et al., 2017; Liew et al., 2018; Poortvliet et al., 2018; Summers et al., 2018; Mamlins et al., 2019), or during the intervention (*n* = 12) (Jayaram et al., 2012; Dutta et al., 2014; Panouillères et al., 2015; Ehsani et al., 2016; Panico et al., 2016; Taubert et al., 2016; Yavari et al., 2016; Samaei et al., 2017; Liew et al., 2018; Summers et al., 2018; Jongkees et al., 2019; Mamlins et al., 2019).

#### Long-Term Motor Learning–Motor Performance After a Break of 24 h or More

Of the five studies which evaluated the effect of ctDCS after a break of 24 h or more, three reported enhanced (Ehsani et al., 2016; Samaei et al., 2017; Jackson et al., 2019), while two reported impaired (Taubert et al., 2016; Jongkees et al., 2019) gains in motor performance with anodal ctDCS. Compared to sham ctDCS, anodal ctDCS enhanced the gains in the performance of a motor skill tasks evaluated after a break of 24 (Jackson et al., 2019) and 48 h (Ehsani et al., 2016; Samaei et al., 2017). This was reflected by a greater reduction in the number of errors and/or faster response time in those aged <40 years (Ehsani et al., 2016; Jackson et al., 2019) and a greater reduction in response time, but not the number of errors, in individuals over 40 years (Samaei et al., 2017). Of the two studies that reported impaired gains in motor performance, one found impaired reaction time, but not the number of errors in a motor skill task after 24 h (Jongkees et al., 2019), and the other reported impaired early adaptation in a motor adaptation task when evaluated after 24 h (Taubert et al., 2016). Two studies evaluated the effect of cathodal ctDCS and found no difference in motor performance 24 h after the intervention (Taubert et al., 2016; Jongkees et al., 2019). These studies applied anodal and cathodal ctDCS centered over the inion (Jongkees et al., 2019) or ipsilateral to the training limb during task training or prior to and in conjunction with task training (Jackson et al., 2019). The stimulation was delivered at a current density of 0.03 mA/cm^2^ (Jongkees et al., 2019) or 0.08 mA/cm^2^ for 20–25 min (Ehsani et al., 2016; Taubert et al., 2016; Samaei et al., 2017; Jackson et al., 2019; Jongkees et al., 2019).

#### Short-Term Motor Learning–Motor Performance After a Break of <24 h

Of the studies that evaluated the effect of anodal ctDCS after a break of <24 h, four found enhanced (Shah et al., 2013; Ehsani et al., 2016; Samaei et al., 2017; Jackson et al., 2019) and one found no effect (Panouillères et al., 2015) on gains in motor performance compared to sham ctDCS. Anodal ctDCS enhanced the performance of a motor skill task by reducing the number of errors but not response time in healthy young individuals (Ehsani et al., 2016) and reduced the response time but not the number of errors in healthy older individuals tested after a break of 35 min (Samaei et al., 2017). Anodal ctDCS also improved performance of motor skill task 5 (Jackson et al., 2019), 10, 30, and 60 min after intervention. All four studies stimulated the lateral cerebellum ipsilateral to the training limb for 15 (Shah et al., 2013), 20 (Ehsani et al., 2016; Samaei et al., 2017), or 25 (Jackson et al., 2019) min at a current density of 0.13 mA/cm^2^ (Shah et al., 2013) or 0.08 mA/cm^2^ (Ehsani et al., 2016; Samaei et al., 2017; Jackson et al., 2019). Whereas anodal ctDCS did not affect the number of errors in a motor adaptation task performed after a gap of 50 min when the stimulation was delivered ipsilateral to the training limb at a current density of 0.06 mA/cm^2^ for 17 min (Panouillères et al., 2015).

One study evaluated the effect of cathodal ctDCS on motor performance after a break of <24 h and reported improvement in ankle tracking accuracy tested after 10, 30, and 60 min (Shah et al., 2013).

#### Immediate Motor Learning–Motor Performance Immediately After the Intervention

Of the studies that evaluated the effect of anodal ctDCS immediately after the intervention, one study reported enhanced (Poortvliet et al., 2018), and five found no effect on gains in motor performance as compared to a sham ctDCS group (Jayaram et al., 2012; Foerster et al., 2017; Liew et al., 2018; Summers et al., 2018; Mamlins et al., 2019). Anodal ctDCS at a current density of 0.03 mA/cm^2^ for 20 min improved the performance by reducing the postural variability and increasing steadiness when the target electrode was placed centrally over the cerebellum (Poortvliet et al., 2018). While the same site of stimulation and current density delivered for 30 min had no effect on finger tracking accuracy (Summers et al., 2018). Anodal ctDCS delivered ipsilateral to the dominant limb at a current density of 0.08 mA/cm^2^ for around 15 min had no effect on static and dynamic balance (Foerster et al., 2017), visuomotor adaptation (Liew et al., 2018; Mamlins et al., 2019), forcefield adaptation (Mamlins et al., 2019), or locomotor adaptation (Jayaram et al., 2012).

Application of cathodal ctDCS had no effect (Mamlins et al., 2019) or impaired (Fernandez et al., 2017; Foerster et al., 2017) gains in motor performance evaluated immediately after stimulation. As compared to sham ctDCS, cathodal ctDCS increased variability in a walking adaptation task (Fernandez et al., 2017) and impaired static but not dynamic balance in adaptation task (Foerster et al., 2017). These effects were seen when ctDCS was delivered ipsilateral to the dominant limb prior to motor task training at a current density of 0.06 mA/cm^2^ (Fernandez et al., 2017) or 0.08 mA/cm^2^ (Foerster et al., 2017) for 20 (Fernandez et al., 2017) or 9 (Foerster et al., 2017) min.

#### Simultaneous Motor Learning–Motor Performance During the Intervention

Application of ctDCS had a varied impact on motor performance during task training. Anodal ctDCS enhanced (*n* = 3) (Jayaram et al., 2012; Ehsani et al., 2016; Yavari et al., 2016), impaired (*n* = 3) (Dutta et al., 2014; Taubert et al., 2016; Jongkees et al., 2019), or had no effect on gains in motor performance during task training (*n* = 5) (Panouillères et al., 2015; Samaei et al., 2017; Liew et al., 2018; Summers et al., 2018; Mamlins et al., 2019). Compared to sham ctDCS, anodal ctDCS enhanced motor performance by improving the rate of adaptation (Jayaram et al., 2012; Yavari et al., 2016) and reduced the number of errors but not response time in a serial reaction time task (Ehsani et al., 2016). These effects were primarily observed when anodal ctDCS was delivered ipsilateral to the dominant limb (Jayaram et al., 2012) or training limb (Ehsani et al., 2016; Yavari et al., 2016) for 15 min (Jayaram et al., 2012; Yavari et al., 2016) or more (Ehsani et al., 2016) at a current density of 0.08 mA/cm^2^. Anodal ctDCS impaired gains in motor performance during a perturbed reaching task (Taubert et al., 2016), visual pursuit task (Dutta et al., 2014), and serial reaction time task (Jongkees et al., 2019). In the serial reaction task, the impaired gains in motor performance occurred in reaction time but not in the number of errors. In the perturbed reaching task, ctDCS was delivered ipsilateral to the training limb for 20 min at a current density of 0.08 mA/cm^2^ (Taubert et al., 2016). Whereas, impaired gains in performance of the serial reaction time task or visual pursuit task were seen when the current was delivered centrally (Jongkees et al., 2019) or on the lateral cerebellum contralateral to the training limb (Dutta et al., 2014) for up to 20 min at a current density of 0.03 mA/cm^2^ (Dutta et al., 2014; Jongkees et al., 2019). Anodal ctDCS had no effect on response time in skill task (Samaei et al., 2017) and the number of errors in adaptation (Panouillères et al., 2015; Liew et al., 2018; Mamlins et al., 2019) or skill task (Summers et al., 2018) when the current density was 0.08, 0.06, and 0.03 mA/cm^2^, respectively. The target electrode was placed either centrally over the cerebellum (Summers et al., 2018) or on the lateral cerebellum ipsilateral to the training limb (Panouillères et al., 2015; Samaei et al., 2017; Liew et al., 2018; Summers et al., 2018; Mamlins et al., 2019) which delivered the stimulation for up to 30 min.

Of the five studies that evaluated the effect of cathodal ctDCS during task training, three reported impaired (Jayaram et al., 2012; Panico et al., 2016; Yavari et al., 2016) and two reported no effects (Jongkees et al., 2019; Mamlins et al., 2019) on gains in motor performance. As compared to sham ctDCS, cathodal ctDCS resulted in impaired adaptation (Jayaram et al., 2012; Panico et al., 2016; Yavari et al., 2016) and impaired rate of de-adaptation (Panico et al., 2016). These effects were seen when cathodal ctDCS was delivered ipsilateral to training limb (Jayaram et al., 2012; Panico et al., 2016; Yavari et al., 2016) for 15 min (Jayaram et al., 2012; Yavari et al., 2016) or more (Panico et al., 2016) at a current density of 0.08 mA/cm^2^. Two studies found no effect of cathodal ctDCS on skill or adaptation task (Jongkees et al., 2019; Mamlins et al., 2019). These studies applied cathodal ctDCS centrally (Jongkees et al., 2019) or ipsilateral to the training limb (Mamlins et al., 2019) during task training alone (Jongkees et al., 2019) or prior to and in conjunction with task training (Mamlins et al., 2019) for up to 20 min at a current density of 0.03 mA/cm^2^ (Jongkees et al., 2019) or 0.08 mA/cm^2^ (Mamlins et al., 2019).

## Discussion

This review aimed to determine the effects of cerebellar transcranial direct current stimulation on motor learning. For the first time, this study provides a systematic review of RCTs to quantify the effects of ctDCS based on the time scale of motor learning. There is a modest body of research, with 17 studies including 629 participants. The body of evidence is subject to considerable risk-of-bias. The main findings of this systematic review are that anodal ctDCS appears to be effective at enhancing motor skill learning in the short (<24 h) and longer-term (≥24 h). Whereas, it appears to have no effect on motor learning immediately after or during stimulation. This review suggests that the type of motor task, the tDCS stimulation parameters and the interaction between task and stimulation parameters are likely to influence the efficacy of ctDCS.

When compared to sham ctDCS, anodal ctDCS appears to be effective at improving short and longer-term motor learning in healthy individuals when applied primarily during motor skill learning (Shah et al., 2013; Ehsani et al., 2016; Samaei et al., 2017; Jackson et al., 2019) but not motor adaptation paradigms (Panouillères et al., 2015; Taubert et al., 2016). Task characteristics and their interaction with the time scale of learning may explain this. Motor skill training paradigms use novel or complex motor skills, which may take weeks or months to master (Schmidt and Lee, 2011). In contrast, motor adaptation tasks involve modifying a well-learnt skill in response to error feedback. Often participants adapt to induced errors within minutes to hours in motor adaptation tasks (Bastian, 2008). It is possible that motor adaptation paradigms are subject to a ceiling effect in healthy individuals. Repeated exposure to the same adaptation task may not provide sufficient stimulus to induce learning (Bastian, 2008; Criscimagna-Hemminger et al., 2010). In addition, an interference task was undertaken between the intervention and testing sessions of one of the motor adaptation tasks, making interpretation of their results challenging (Taubert et al., 2016).

The reported gains in the performance of a motor skill task in response to anodal ctDCS may also depend on the measure of motor performance used and the age of the participants. In studies investigating healthy young individuals undertaking a unimanual serial reaction time task, ctDCS enhances accuracy but not response time after a break of <24 h and enhanced accuracy and response time after a break of 24 h or more (Ehsani et al., 2016). A previous non-randomized experimental study has also reported that ctDCS may have a greater effect on accuracy than response time within and after 24 h (Cantarero et al., 2015). In contrast, in a study investigating healthy older individuals undertaking the same task, a greater reduction in response time but not the number of errors was observed in response to ctDCS irrespective of the time scale of measurement (Samaei et al., 2017). These findings suggest that ctDCS may differentially influence short and longer-term motor learning of different parameters of movement performance. However, it is unclear whether the difference between older and younger individuals reflects differences in the mechanism of action of ctDCS or that older individuals have slower response time but not greater inaccuracy in these types of task (Voelcker-Rehage, 2008).

In studies which investigated the effects of ctDCS using serial reaction time tasks, conflicting results were observed. Improved response times were seen in a unimanual task (Ehsani et al., 2016; Samaei et al., 2017), whereas impaired reaction time was seen in a bimanual task (Jongkees et al., 2019). The performance measure used to reflect motor learning in the two tasks may evaluate different aspects of motor performance. Reaction time reflects the time between stimulus appearance and movement initiation. Whereas, response time is comprised of both reaction time and movement time (Pascual-Leone et al., 1995). However, it is notable that the studies also differed in the stimulation parameters used, where a current density of 0.03 mA/cm^2^ centered over bilateral cerebellar hemisphere impaired gains, while a current density of 0.08 mA/cm^2^ targeting the lateral cerebellum ipsilateral to the training limb enhanced gains in motor performance. The challenge of unpacking these conflicting results illustrates the importance of taking a systematic approach to investigating ctDCS; where the influence of motor task, performance metric, and stimulation parameters should be considered.

Anodal ctDCS appears to have no effect on gains in motor performance measured during and immediately after the intervention, where most of the studies demonstrated no effect (Jayaram et al., 2012; Panouillères et al., 2015; Foerster et al., 2017; Samaei et al., 2017; Liew et al., 2018; Summers et al., 2018; Mamlins et al., 2019) and some enhanced (Jayaram et al., 2012; Ehsani et al., 2016; Yavari et al., 2016; Poortvliet et al., 2018) or impaired (Dutta et al., 2014; Taubert et al., 2016; Jongkees et al., 2019) gains in motor performance. These results were observed irrespective of the type of task being studied (adaptation or skill) as has been noted in previous narrative reviews (Ferrucci et al., 2015; van Dun et al., 2016). It is therefore unclear whether ctDCS has any effect on motor learning during or immediately after task training. Motor learning research highlights the paradoxical relationship between learning and performance. That is, motor learning, as defined as a permanent change in motor performance, can occur without immediate changes in motor performance. In fact, immediate changes in motor performance in response to an intervention are often not sustained after a break (Soderstrom and Bjork, 2015). This suggests that changes in motor performance during and immediately after anodal ctDCS are less relevant in determining the effectiveness of anodal ctDCS than changes observed after 24 h or more.

This systematic review highlights that the site of anodal ctDCS stimulation and current density are the critical stimulation parameters which appear to impact the effect produced, irrespective of time scale. Greater gains in motor performance were seen with the target electrode placed centrally on the cerebellum in a bilateral postural control task (Poortvliet et al., 2018) and ipsilateral to the training limb in unilateral tasks (Shah et al., 2013; Ehsani et al., 2016; Samaei et al., 2017). In addition, motor performance is enhanced during a bilateral task involving greater perturbation to one of the limbs with the placement of target electrode ipsilateral to that limb (Jayaram et al., 2012). This suggests that the parameters of the motor task may be an important consideration in determining an appropriate site for stimulation. Therefore, researchers should explicitly consider where in the cerebellum motor control and learning is occurring for a given task and select electrode configuration with this in mind (Hulst et al., 2017), acknowledging that current density and specificity is dependent on electrode size and position (Ferrucci et al., 2013). Positive effects were more likely to be observed when anodal ctDCS was delivered with a current density of 0.08 mA/cm^2^ or more. This current density is greater than that recommended for cerebral ctDCS (Nitsche et al., 2003); however, modeling studies illustrate the need for higher current density to stimulate the cerebellum to overcome large shunting of current at the base of the skull (Rampersad et al., 2014). Other stimulation parameters such as stimulation duration and timing of stimulation delivery (at rest or during task training) had an equivocal effect. The total duration of stimulation was not hugely variable and ranged from 15 to 20 min. Contrary to previous literature (Monte-Silva et al., 2010), no relationship between stimulation duration and time scale of effect was observed. Further research is required to unpack the effect of stimulation duration on the permanence of ctDCS effects across time scales.

When compared to sham ctDCS, cathodal ctDCS has an equivocal effect on short and longer-term motor learning in healthy individuals. However, most of the studies found impaired gains in motor performance of adaptation tasks during and immediately after cathodal ctDCS (Jayaram et al., 2012; Panico et al., 2016; Yavari et al., 2016; Fernandez et al., 2017; Foerster et al., 2017) with few reporting no effect on gains in motor performance (Jongkees et al., 2019; Mamlins et al., 2019). Overall, there is insufficient evidence to infer the effect of cathodal ctDCS on motor learning.

Although most of the included studies employed randomized, blinded, sham-controlled designs, their methodological quality was globally considered to have “high” risk-of-bias. Potential sources of bias included failure to report the method of randomization used, allocation concealment and failure to explicitly state who was blinded: the participant, the person administering the intervention, and/or the outcome assessor. The majority of studies did not report trial registration details or a pre-specified statistical analysis plan. Further, some studies had baseline differences between intervention groups that suggested a problem with the randomization process. Whilst these judgments of research quality may not reflect what the researchers actually did during the protocol but rather a lack of explicit documentation; it is essential that adherence to, and reporting of, these standards of practice become commonplace in this body of literature. The potential for bias may contribute to the reporting of contradictory results and suggests that the interpretation of the research findings to date must be approached with some caution (Steiner et al., 2016; Hulst et al., 2017; Jalali et al., 2017).

### Limitations, Implications and Future Research

The included studies had considerable variability in both measurement and data processing methods. Some studies measured the time course of change in error throughout the task training (Panouillères et al., 2015), some in specific epochs (early or late epochs) (Panico et al., 2016; Taubert et al., 2016), some fitted an exponential curve (Jayaram et al., 2012; Yavari et al., 2016), while other measured change scores (Shah et al., 2013; Ehsani et al., 2016; Samaei et al., 2017; Jackson et al., 2019). Furthermore, the method for calculating changes in motor performance was inconsistent across studies. For instance, the error was calculated as mean error (Jayaram et al., 2012), mean absolute error (Dutta et al., 2014), or normalized accuracy index using root mean square error (Shah et al., 2013) while others failed to describe how the error was calculated (Ehsani et al., 2016). The method by which error is calculated affects its accuracy; for example, a simple mean of errors may not reflect individual variability while a mean absolute error encompasses bias due to individual variability (Schmidt and Lee, 2011). This makes comparing results across studies challenging.

Despite these limitations, the review adds to our understanding of the potential of ctDCS to impact motor learning, with particular reference to the time scale of learning. It highlights the importance of task characteristics, movement parameter outcome measurement techniques, participant age, and stimulation parameters when interpreting the research body and designing future studies. Further research, which explores the time scales of >24 h are required. There are also many unanswered questions regarding the cumulative effects of ctDCS over multiple sessions and the long-term retention of performance after a delay of weeks and months. More studies evaluating the effect of ctDCS on motor adaptation tasks over longer time scales are needed to elucidate its effect on adaptive learning.

## Conclusions

In conclusion, anodal ctDCS appears to be effective at improving short and long-term motor skill learning. However, these results are predicated upon just four modest-quality studies. While these findings illustrate the potential of targeting the cerebellum with tDCS to enhance learning in healthy and clinical populations, researchers need to take a methodologically robust and systematic approach to future research. Factors including the challenge of the motor task and its characteristics, the ctDCS stimulation parameters, method of measuring motor performance, and participant age are likely to influence whether ctDCS will enhance or have no effect on motor learning.

## Data Availability Statement

The raw data supporting the conclusions of this manuscript will be made available by the authors, without undue reservation, to any qualified researcher.

## Author Contributions

All authors were involved in the conceptualization and designing of the study. NK was involved with the literature search and data extraction. NK and NS were involved with manuscript preparation. NS and DT were involved in supervision. All authors were involved in reviewing and editing the manuscript.

### Conflict of Interest

The authors declare that the research was conducted in the absence of any commercial or financial relationships that could be construed as a potential conflict of interest.
