# Supplementary material for: The Effect of Cerebellar Transcranial Direct Current Stimulation on Motor Learning: A Systematic Review of Randomized Controlled Trials
Source: Front Hum Neurosci. 2019 Oct 4;13:328. doi: 10.3389/fnhum.2019.00328 (PMC6788395; doi:10.3389/fnhum.2019.00328)
Supplement: Supplementary file 1 [file Data_Sheet_1.docx]

Supplementary File 1: Search terms, search strategy.

| **AND** | **Search Terms** |
| --- | --- |
| Motor learning | acquisition  "motor performance"  OR  "motor control"  learning  adapt* |
| Cerebellar tDCS | ctDCS  OR  "cerebellar stimulation"  tDCS  OR  "transcranial direct current stimulation"  OR  "non-invasive brain stimulation"  "noninvasive brain stimulation"  AND  "direct current stimulation"  cerebell* |

* truncation format used in the specific database
