# Supplementary figures and images for: The Effect of Cerebellar Transcranial Direct Current Stimulation on Motor Learning: A Systematic Review of Randomized Controlled Trials
Source: Front Hum Neurosci. 2019 Oct 4;13:328. doi: 10.3389/fnhum.2019.00328 (PMC6788395; doi:10.3389/fnhum.2019.00328)

Supplementary File 2 Quality of included studies.


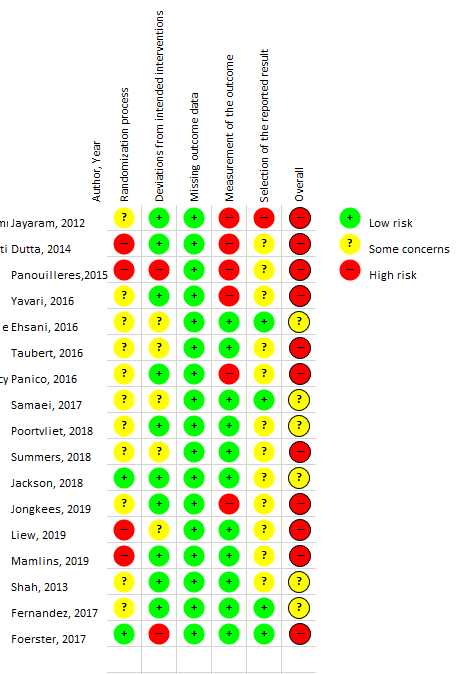

Supplement: Supplementary file 2 [file Data_Sheet_2.docx]
